# Nontargeted Metabolomics for Phenolic and Polyhydroxy Compounds Profile of Pepper (*Piper nigrum* L.) Products Based on LC-MS/MS Analysis

**DOI:** 10.3390/molecules23081985

**Published:** 2018-08-09

**Authors:** Fenglin Gu, Guiping Wu, Yiming Fang, Hongying Zhu

**Affiliations:** 1 Spice and Beverage Research Institute, Chinese Academy of Tropical Agricultural Sciences, Wanning 571533, China; guiping81@163.com (G.W.); fym870902@163.com (Y.F.); 2 National Center of Important Tropical Crops Engineering and Technology Research, Wanning 571533, China; 3 Key Laboratory of Genetic Resources Utilization of Spice and Beverage Crops, Ministry of Agriculture, Wanning 571533, China

**Keywords:** pepper (*Piper nigrum* L.) products, nontargeted metabolomics, phenolic and polyhydroxy compounds, principal component analysis (PCA)

## Abstract

In the present study, nontargeted metabolomics was used to screen the phenolic and polyhydroxy compounds in pepper products. A total of 186 phenolic and polyhydroxy compounds, including anthocyanins, proanthocyanidins, catechin derivatives, flavanones, flavones, flavonols, isoflavones and 3-*O*-*p*-coumaroyl quinic acid *O*-hexoside, quinic acid (polyhydroxy compounds), etc. For the selected 50 types of phenolic compound, except malvidin 3,5-diglucoside (malvin), l-epicatechin and 4′-hydroxy-5,7-dimethoxyflavanone, other compound contents were present in high contents in freeze-dried pepper berries, and pinocembrin was relatively abundant in two kinds of pepper products. The score plots of principal component analysis indicated that the pepper samples can be classified into four groups on the basis of the type pepper processing. This study provided a comprehensive profile of the phenolic and polyhydroxy compounds of different pepper products and partly clarified the factors responsible for different metabolite profiles in ongoing studies and the changes of phenolic compounds for the browning mechanism of black pepper.

## 1. Introduction

Various metabolic profiling tools have been widely used in metabolomics. Nuclear magnetic resonance (NMR) spectroscopy is the primary tool utilized for metabolic profiling [1]. In addition to NMR, liquid chromatography-mass spectrometry (LC-MS) has also been used for metabolic profiling, and numerous LC-MS applications have been reported in the analyses of hydrophobic and hydrophilic metabolites [2,3,4,5].

Pepper (*Piper nigrum* Linnaeus) is a major commercial spice that is valued for its pungency and flavor. Black, white, and green peppers are the three main types of pepper products on the market. For white pepper, the traditional processing method is picking the mature pepper fruit that had dried after being soaked in water and peeled. Black pepper (*Piper nigrum* L.) is the most widely used spice worldwide and is an important health food. Fresh pepper fruits are traditionally exposed to sunlight for three to four days on the bleachery. When peels are wrinkled and shrunken, the fruit stems are removed, and the fruits are fully sun-dried 1 to 2 days. Direct solarization is a simple and cheap method that is still widely adopted in pepper-producing countries. Blanching refers to the process in which pepper berries are soaked in water at 80 °C for 2 or 3 min and then solarized. Blanching removes dust, insects, and microorganisms from the surface of pepper berries, helps the stemming of peppers, and accelerates the drying speed of peppers, resulting in increasingly black color and lustrous appearance. However, studies have demonstrated that blanching can damage the cellular structure of pepper, leading to rapid water loss. At the same time, polyphenol oxidase in different areas can combine with the polyphenol substrate and promote blackening [6].

Blackening is beneficial and important in black pepper processing given its contribution to the color and flavor of black pepper. Blackening of pepper can be achieved using two methods, namely, enzymatic blackening and non-enzymatic blackening. Variyar et al. found that the blackening of pepper berries was mainly caused by the catalytic oxidation of the glucoside compound 3,4-dihydroxyphenylethanol and its aglycone by pyrocatechase [7]. Bandyopadhyay et al. studied the natural drying process of fresh pepper fruitfruits of pepper berries. They found that the total phenolic content decreased by approximately 75%, whereas phenolic substance oxidized by *O*-diphenolase completely disappeared. This finding indicated that phenolic compounds were major contributors to blackening in the preparation of black pepper. However, no consistent relationship was observed between the phenolic content that can be oxidized by pyrocatechase and the blackening degree [8]. These observations suggest that the blackening of black pepper was due to other mechanisms and that the black substances formed by other routes strongly influence the color, luster, and flavor of black pepper. These findings and other reports provide a scientific basis for the necessity of our study [9,10,11,12].

Our previous studies found that heat treatment deepened the color of pepper berries, but polyphenol oxidase activity significantly decreased during the preparation of black pepper [13]. In addition, the concentrations of phenolic compounds, vitamin C and chlorophyll a and b also significantly decreased. Polyphenol oxidation and chlorophyll and vitamin C degradation contribute to blackening [14].

Herein we selected 15 samples, which were divided into five groups for metabolic analysis. Three biological replicates were prepared for each group. The metabolic differences of the groups were assessed by combining a liquid chromatography-tandem mass spectrometry (LC-MS/MS) detection platform, a self-built database, and multivariate statistical analysis. This study aimed to further discuss the phenolic compound involved in the blackening during pepper processing to reveal the mechanism underlying pepper blackening.

## 2. Results and Discussion

### 2.1. Evaluation of the Repetition Correlation

**Figure 1:**
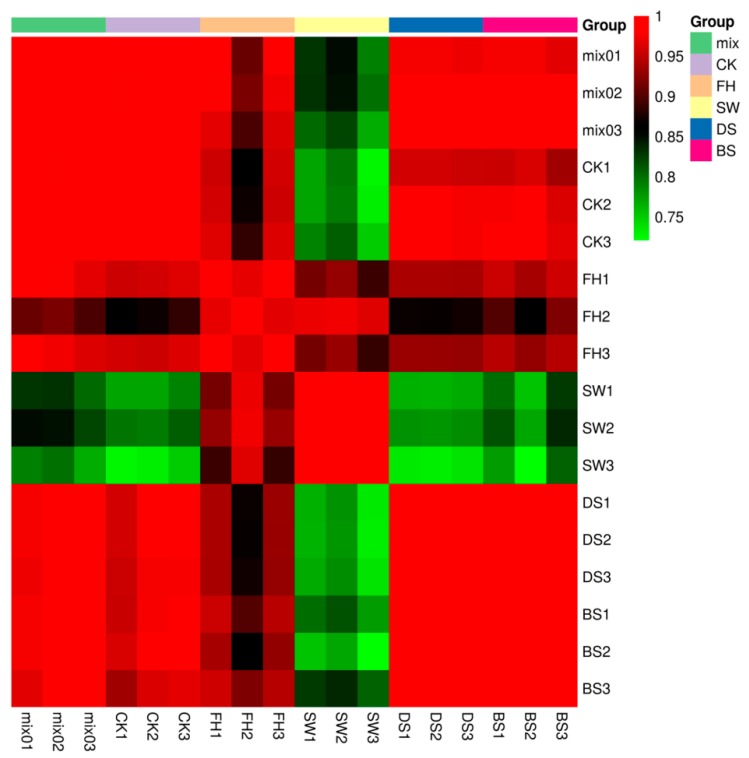
Repetitive correlation between samples of different groups (CK, BS, DS, FH, and SW are described in the sample information construction method) and the quality control sample (mix: mixture of extractive of samples).

Biological replicates were obtained among samples in the group by performing correlation analysis between samples. High correlation coefficient of the samples in the group relative to the intergroup samples indicated a reliable metabolite. The Pearson’s correlation coefficient r was considered the evaluation indicator for the correlation of biological replicates. An r^2^ value approaching 1 indicates strong correlation between two repetitive samples. Figure 1 shows excellent intra-group repetitiveness. The experimental data ensured the accuracy of analysis results. Good inter-group repetitiveness was observed except in white pepper. The low repetitiveness of white pepper may be due to its preparation using the water-soaking method. Thus, in terms of microbial action during pepper soaking and decrustation, the phenolic compounds may display significant variations [8].

### 2.2. Identification and Comparison of Phenolic and Polyhydroxy Compounds 

The first step in the experimental procedures was to gather information on several compounds in pepper samples by non-targeted analysis. The non-targeted LC-MS/MS method identified 186 phenolic and polyhydroxy compounds in five different types of pepper product samples (Table 1). Anthocyanins, proanthocyanidins, catechin derivatives, flavanones, flavones, flavonols, isoflavones, flavone *C*-glycosides, hydroxycinnamoyl derivatives, quinate and its derivatives, and flavonolignan were detected in the first overview of the results.

**Figure 2:**
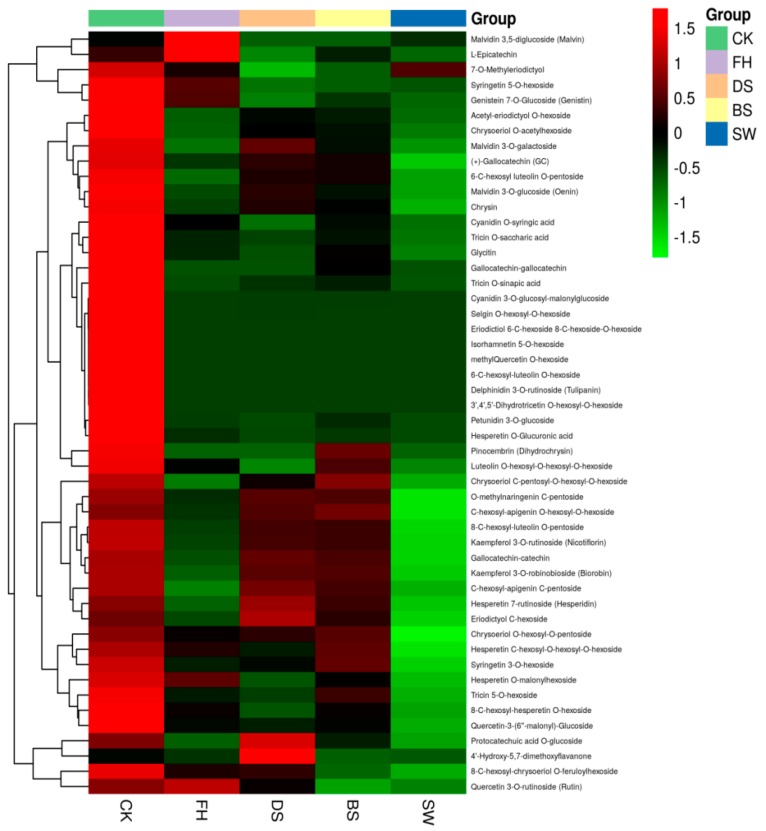
Intensity (in logarithmic scale) of the metabolites in the different pepper samples visualized as a heat map. The dendrogram represents the hierarchical clustering of the samples.

Among the 186 compounds, 50 compounds with significant changes were selected for further analysis, as shown in Figure 2. The overall profiles of phenolic and polyhydroxy compounds varied among the five samples. In the freeze-dried fresh berries samples, the relative contents of other 47 substances (excluding malvidin 3,5-diglucoside (malvin), l-epicatechin, and 4′-hydroxy-5,7-dimethoxyflavanone) were relatively high. In the FH sample, malvidin 3,5-diglucoside (malvin), l-epicatechin, quercetin 3-*O*-rutinoside (rutin), and hesperetin *O*-glucuronic acid had relatively high contents. Hrazinda et al. described the stability of malvidin 3,5-diglucoside (malvin) and found that compared with 3,5-diglucosides of cyanidin, peonidin, delphinidin, and petunidin, malvidin-3,5-diglucoside was the most stable, followed by peonidin, petunidin, cyaniclin, and delphinidin-3,5-diglucosides [15]. Therefore, the relative contents in the FH samples were relatively high. However, in the DS and BS samples, the content of malvidin 3,5-diglucoside (malvin) was relatively low possibly due to thermal treatment, solarization, oxidation degradation, and thermal degradation of anthocyanins [16]. Other derivatives were likely generated. As shown in Figure 2, the relative contents of malvidin 3-*O*-glucoside (oenin) and malvidin 3-*O*-galactoside were higher in the DS sample compared with other samples. Compared with those in FH samples, the relative content of l-epicatechin in CK samples was low, but its content was higher than that in the DS, BS, and SW samples. This finding is possibly due to the existence of the compound in the peels. Given the short time of the hot air drying and the lack of blanching and peeling, the content was relatively high. Liu et al. investigated the degradation mechanism of cyanidin 3-rutinoside in the presence of (−)-epicatechin and litchi pericarp polyphenol oxidase. They presented that the enzymatic oxidation of (−)-epicatechin produced the corresponding *O*-quinone. Then, cyanidin 3-rutinoside and (−)-epicatechin competed for (−)-epicatechin *O*-quinone, resulting in the degradation of cyanidin 3-rutinoside and regeneration of (−)-epicatechin [17]. In DS and BS samples, *O*-methylnaringenin *C*-pentoside, kaempferol 3-*O*-robinobioside (biorobin), pinocembrin (dihydrochrysin), and kaempferol 3-*O*-rutinoside (nicotiflorin) were present at relatively high contents. Pinocembrin is a novel natural compound with versatile pharmacological and biological activities [18]. 

In black pepper prepared by solarization after blanching, such substance is relatively abundant. Therefore, the black pepper prepared in this manner showed considerable activity. Substances such as kaempferol 3-*O*-robinobioside (biorobin) and kaempferol 3-*O*-rutinoside (nicotiflorin) exhibited moderate antioxidant activities [19]. Thus, only a small amount of these compounds was oxidized during solar drying, and their relative contents were relatively high. 4′-Hydroxy-5,7-dimethoxyflavanone was first reported in pepper.

### 2.3. Comparison of Phenolic and Polyhydroxy Compounds Content

**Figure 3:**
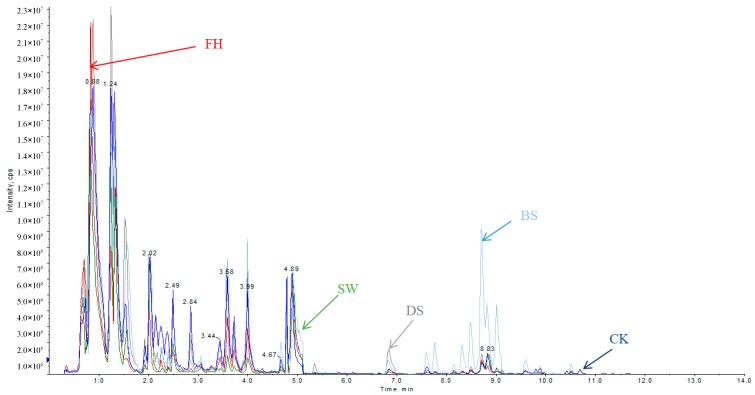
LC-MS/MS base peak intensity (BPI) profiles of the phenolic and polyhydroxy compounds of samples (CK, BS, DS, FH, and SW are described in the sample information construction method).

Changes in the LC-MS/MS base peak intensity (BPI) chromatogram profiles of the phenolic and polyhydroxy compounds of samples are shown in Figure 3. The profiles were remarkably different among the five kinds pepper products we investigated. The fresh pepper berries (CK) exhibited the most abundant phenolic and polyhydroxy compounds, and the total peak area was the highest, while the peaks of the white pepper (SW) were the lowest. Specifically, fresh pepper fruits exposed to heat treatment with hot water for 2 min at 80 °C and dried under the sun, large quantities of hydrophobic compounds were produced. As can be seen in Figure 3, the black pepper of the blanching for fresh pepper fruit and sun-dried (BS) owned the different composition from other four kinds of pepper products, and the black substances formed by other routes strongly influence the color, luster, and flavor of black pepper [7,8,9]. This figure (Figure 3) increases the understanding of the phenolic and polyhydroxy constitution and changes including the relative content of phenolic and polyhydroxy compounds in different pepper samples. This study provides many interesting findings worth of further investigation.

### 2.4. Principal Component Analysis (PCA)

**Figure 4:**
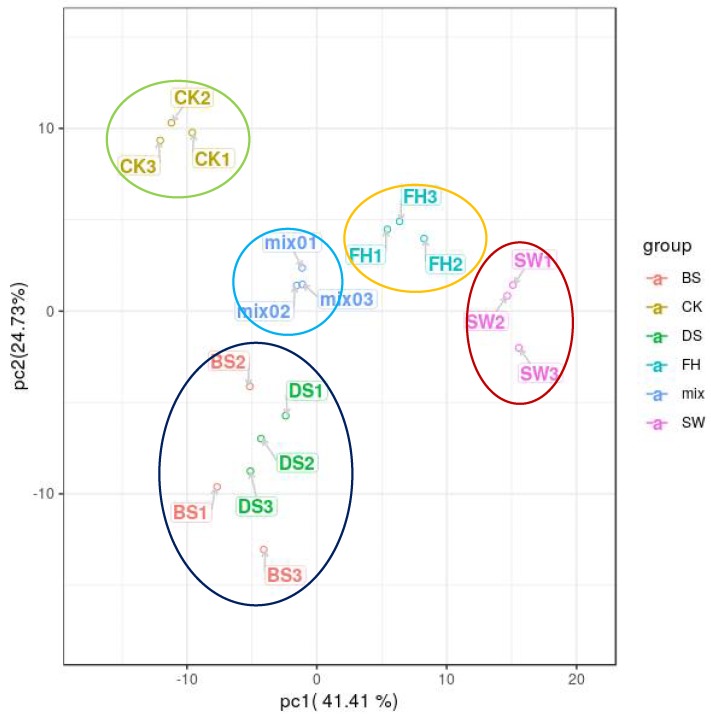
PCA scores of samples and the quality control sample (CK, BS, DS, FH, and SW are described in the sample information construction method and mix is the mixture of extractive of samples).

PCA was performed to develop a visual plot to evaluate the resemblance and difference in metabolic profiles of pepper samples on the basis of 186 significant altered metabolites. Based on the Scree plot of cumulative eigenvalues (Figure 4), we selected two PCs that explained 66.14% of variations among phenolic and polyhydroxy components. PC1 and PC2 can explain 41.41% and 24.73% of the sample difference. PCA analysis of polyphenol and polyhydroxy compounds in the sample revealed that the samples can be divided to four groups: freeze-dried pepper, hot-air-dried pepper, white pepper, and black pepper. The method for processing the pepper samples suggests that the polyphenol and polyhydroxy compounds in the CK may be the most abundant components [8]. Polyphenol and polyhydroxy compounds showed the lowest contents in the SW sample (white pepper). Polyphenol and polyhydroxy compounds were present at moderate contents in FH (hot-air-dried) and the lowest contents in BS and DS (black pepper). These findings were consistent with our previous conclusion on the preparation of phenolic compounds in black pepper. DS and BS samples can be assigned to one group, indicating that the polyphenol and polyhydroxy compounds were highly similar in terms of type and content.

## 3. Experimental Section 

### 3.1. Materials and Chemicals

Pepper berries were harvested from the plant garden of the Spice and Beverage Research Institute, Chinese Academy of Tropical Agricultural Sciences (Haikou, China). Harvesting commenced when one or two berries per spike had turned orange or red. The fresh pepper berries were then freeze-dried (named as CK), oven-dried at 50 °C (named as FH), soaked in flowing water for 5 days for white pepper preparation (named as SW), dried directly under the sun for seven days (named as DS), exposed to heat treatment with hot water for 2 min at 80 °C and dried under the sun (named as BS). Treatment was continued until the moisture content reached 8%–12%. All reagents used in the L*C*-MS/MS analyses were of HPLC grade. HPLC-grade acetonitrile, methanol, and ethanol were purchased from Merck KGaA (Darmstadt, Germany). Other standard chemicals were purchased from BioBioPha Co. (Kunming, Yunnan, China) or Sigma-Aldrich Co. (St. Louis, MO, USA). The standard substance was dissolved in dimethyl sulfoxide or methanol and then stored at −20 °C. Distilled water was purified using a Millipore Milli-Q20 System (Bedford, MA, USA).

### 3.2. Extraction and Separation of Phenolic and Polyhydroxy Metabolites

Freeze-dried samples were crushed using a mixer mill (MM 400, Retsch, Haan, Germany) with a zirconia bead for 1.5 min at 30 Hz. Exactly 100 mg of powder was weighed and extracted overnight at 4 °C with 1.0 mL of 70% aqueous methanol. After centrifugation at 10,000× *g* for 10 min, the extracts were adsorbed (CNWBOND Carbon-GCB SPE Cartridge, 250 mg, 3 mL; ANPEL, Shanghai, China) and filtrated (SCAA-104, 0.22 μm pore size; ANPEL) prior to LC–MS/MS analysis. A quality control sample was prepared by equally blending all samples. During the assay, the quality control sample was run every 10 injections to monitor the stability of analytical conditions.

Samples (5 μL) were injected into an HPLC system (Shimpack UFLC Shimadzu CBM30A, Kyoto, Japan) consisting of a binary pump, an online vacuum degasser, an autosampler, and a column compartment. Phenolic compounds were separated on a Waters ACQUITY UPLC HSS T3 C18 (1.8 µm, 2.1 mm × 100 mm) (Milford, MA, USA) at 40 °C. Mobile phase A was water containing 0.04% acetic acid, and mobile phase B was acetonitrile containing 0.04% acetic acid. The flow rate was 0.4 mL/min. The gradient profile was as follows: 5% B at 0 min; linear gradient to 95% B from 0 min to 11 min; isocratic 95% B from 11 min to 12 min; linear gradient to 5% B from 12 min to 12.1 min; and isocratic 95% B from 12.1 min to 15 min. The injection volume of the standard solutions and samples was 2 μL. After each injection, the needle was rinsed with 600 μL of weak wash solution (water/methanol: 90:10) and 200 μL of strong wash solution (methanol/water: 90:10). The samples were maintained at 6 °C during analysis.

### 3.3. Metabolite Identification and Quantification

Metabolites were identified on a 6500 QTRAP system (Applied Biosystems, Foster City, CA, USA) instrument equipped with an electrospray source. Capillary voltages were 5.5 and −2.5 kV under positive and negative modes, respectively. The source was maintained at 550 °C. Desolvation temperature, cone gas flow, and desolvation gas flow were 500 °C, 50 L/h, and 800 L/h, respectively. Unit resolution was applied to each quadrupole. The flow injections of each metabolite were used to optimize multiple reaction monitoring (MRM) conditions. For most metabolites, this step was performed according to the method reported by Chen et al. [20].

Qualitative analysis was performed on the data of the first-order spectra and the self-built database obtained by mass spectrum tests based on a self-built database MWDB (MetWare database) and the metabolite information common database. Among the metabolites, the several substances have already been identified, and the repetitive signals of the adducts containing ions of K^+^, Na^+^, and NH_4_^+^ and the mono-isotopic signal were removed during analysis. The repetitive signals of fragment ions with large molecular weights were also removed. For the structural analysis of metabolites, we referred to MassBank (http://www.massbank.jp/), KNAPSAcK (http://kanaya.naist.jp/KNApSAcK/), HMDB (http://www.hmdb.ca/), MoTo DB (http://www.ab.wur.nl/moto/), METLIN (http://metlin.scripps.edu/index.php) [21] and other existing mass spectrum common databases. Metabolite intensity was conducted using MRM. Partial least squares discriminant analysis was conducted on the identified metabolites. Metabolites with significant differences in content were set with thresholds of variable importance in projection (VIP) ≥ 1 and fold change ≥ 2 or ≤ 0.5.

## 4. Conclusions 

In this study, metabolomics was adopted to analyze the composition of phenols and polyhydroxy compounds in pepper products prepared by five common methods to prove the influence of the pepper processing method on the composition, color, and flavor of pepper. This study also aimed to indirectly verify previous findings indicating that phenolic substances directly participate in blackening without enzymatic reaction. Given that decrustation was employed in this work, the phenolic substance content in white pepper was relatively low. However, for black pepper prepared by blanching and drying and black pepper prepared by direct solarization, the basic composition and content of phenols displayed slight differences. Pinocembrin, which was detected from black pepper prepared by blanching, exhibited multiple biological activities, including antimicrobial, anti-inflammatory, antioxidant, and anticancer. This substance can also be used as a neuroprotective agent against cerebral ischemic injury with a wide therapeutic time window. These findings provide good references for further studies on the processing of black pepper.

## Figures and Tables

**Table 1:** 186 Metabolites detected in pepper products by LC-MS/MS systems.

**Anthocyanins**					
1. Pelargonidin	2. Malvidin 3,5-diglucoside (Malvin)	3. Cyanidin *O*-syringic acid	4. Cyanidin 3-*O*-glucoside (Kuromanin)	5. Malvidin 3-*O*-glucoside (Oenin)	6. Malvidin 3-*O*-galactoside
7. Peonidin *O*-hexoside	8. Petunidin 3-*O*-glucoside	9. Cyanidin 3-*O*-glucosyl-malonylglucoside	10. Delphinidin 3-*O*-rutinoside (Tulipanin)		
**Catechin Derivatives**					
11. 4-Methylcatechol	12. Epigallocatechin (EGC)	13. Epicatechin gallate (ECG)	14. (+)-Gallocatechin (GC)	15. Catechin-catechin-catechin	16. Gallocatechin-gallocatechin
17. Protocatechuic acid	18. Gallocatechin-catechin	19. Catechin	20. l-Epicatechin	21. Protocatechuic aldehyde	22. Protocatechuic acid *O*-glucoside
**Proanthocyanidins**					
23. Procyanidin A2	24. Procyanidin A1	25. Procyanidin B2	26. Procyanidin B3		
**Flavanones**					
27. Naringenin chalcone	28. Afzelechin (3,5,7,4′-Tetra-hydroxyflavan)	29. Isosakuranetin (4′-Methyl-naringenin)	30. Hesperetin	31. Homoeriodictyol	32. Naringenin
33. Eriodictyol	34. Pinocembrin (Dihydrochrysin)	35. Naringenin 7-*O*-glucoside (Prunin)	36. 7-*O*-Methyleriodictyol	37. Butein	38. Naringenin 7-*O*-neohesperidoside (Naringin)
39. Hesperetin 7-*O*-neo-hesperidoside (Neohesperidin)	40. 4′-Hydroxy-5,7-dimethoxyflavanone	41. Hesperetin 7-rutinoside (Hesperidin)			
**Flavones**					
42. Chrysoeriol *O*-rhamnosyl-*O*-glucuronic acid	43. Baicalein (5,6,7-Trihydroxyflavone)	44. Acacetin	45. Tricin	46. Butin	47. Tricin *O*-hexosyl-*O*-syringin alcohol
48. Luteolin	49. 7,4′-Dihydroxyflavone	50. Acetyl-eriodictyol *O*-hexoside	51. Luteolin *O*-hexosyl-*O*-hexosyl-*O*-hexoside	52. Limocitrin *O*-hexoside	53. Apigenin *O*-malonylhexoside
54. Chrysoeriol	55. Apigenin *O*-hexosyl-*O*-rutinoside	56. Apigenin	57. Tangeretin	58. Chrysoeriol *O*-hexosyl-*O*-pentoside	59. Apigenin 7-*O*-glucoside (Cosmosiin)
60. Apigenin 5-*O*-glucoside	61. Luteolin *O*-hexosyl-*O*-pentoside	62. Tricin 7-*O*-hexoside	63. Nobiletin	64. Chrysoeriol *O*-acetylhexoside	65. Syringetin 5-*O*-hexoside
66. Luteolin 3′,7-di-*O*-glucoside	67. Apigenin *O*-hexosyl-*O*-pentoside	68. Chrysin	69. Chrysoeriol 7-*O*-hexoside	70. Chrysoeriol 5-*O*-hexoside	71. Chrysoeriol 7-*O*-rutinoside
72. Tricin 5-*O*-hexoside	73. 3′,4′,5′-Dihydrotricetin *O*-hexosyl-*O*-hexoside	74. Luteolin 7-*O*-glucoside (Cynaroside)	75. Tricin *O*-sinapic acid	76. Velutin	77. Luteolin *O*-sinapoylhexoside
78. Tricin *O*-saccharic acid	79. Selgin *O*-hexosyl-*O*-hexoside	80. Apigenin 7-rutinoside (Isorhoifolin)	81. Apigenin 7-*O*-neohesperidoside (Rhoifolin)		
**Flavonols**					
82. Kaempferol 7-*O*-rhamnoside	83. Syringetin	84. Morin	85. Kumatakenin	86. Quercetin 3-*O*-rutinoside (Rutin)	87. Kaempferol 3-*O*-rhamnoside (Kaempferin)
88. Kaempferol 3,7-dirhamnoside (Kaempferitrin)	89. Quercetin 7-*O*-rutinoside	90. Quercetin	91. Ayanin	92. Isorhamnetin	93. Myricetin
94. Dihydroquercetin (Taxifolin)	95. Kaempferol 3-*O*-glucoside (Astragalin)	96. Syringetin 3-*O*-hexoside	97. Ethylquercetin *O*-hexoside	98. Aromadedrin Dihydro-kaempferol)	99. Kaempferol-3-*O*-robinoside-7-*O*-rhamnoside (Robinin)
100. Isorhamnetin 5-*O*-hexoside	101. Quercetin-3-(6″-malonyl)-Glucoside	102. Kaempferol 3-*O*-galactoside (Trifolin)			
**Isoflavones**					
103. Biochanin A	104. Orobol (5,7,3′,4′-tetra-hydroxyisoflavone)	105. Daidzein	106. Rotenone	107. 2′-Hydroxygenistein	108. Formononetin (4′-*O*-methyldaidzein)
109. Genistein 7-*O*-Glucoside (Genistin)	110. Glycitin				
**Flavone *C*-glycosides**					
111. Chrysin *C*-hexoside	112. Apigenin *C*-hexosyl-*O*-rutinoside	113. Chrysoeriol *C*-pentosyl-*O*-hexosyl-*O*-hexoside	114. Chrysoeriol 8-*C*-hexoside	115. Apigenin 6-*C*-pentoside	116. Apigenin 8-*C*-pentoside
117. Hesperetin *C*-hexosyl-*O*-hexosyl-*O*-hexoside	118. Eriodictyol *C*-hexoside	119. *O*-methyl-naringenin *C*-pentoside	120. Naringenin *C*-hexoside	121. *C*-hexosyl-chrysin *O*-feruloylhexoside	122. 6-*C*-hexosyl luteolin *O*-pentoside
123. Eriodictiol 6-*C*-hexoside 8-*C*-hexoside-*O*-hexoside	124. Luteolin *C*-hexosyl-*O*-rhamnoside *O*-hexoside	125. *C*-Hexosyl-apigenin *C*-pentoside	126. 6-*C*-Hexosyl-luteolin *O*-hexoside	127. *C*-Hexosyl-chrysoeriol *O*-hexoside	128. di-*C*, *C*-Hexosyl-luteolin
129. 8-*C*-Hexosyl-luteolin *O*-pentoside	130. Luteolin *C*-hexoside	131. 8-*C*-Hexosyl-chrysoeriol *O*-feruloylhexoside	132. Apigenin *C*-glucoside	133. 8-*C*-Hexosyl-apigenin *O*-hexosyl-*O*-hexoside	134. 8-*C*-Hexosyl-apigenin *O*-feruloylhexoside
135. *C*-Pentosyl-chrysoeriol 7-*O*-feruloylhexoside	136. Luteolin 8-*C*-hexosyl-*O*-hexoside	137. *C*-Hexosyl-apigenin *O*-hexosyl-*O*-hexoside	138. *C*-Pentosyl-*C*-hexosyl-apigenin	139. Chrysoeriol *C*-hexosyl -*O*-rhamnoside	140. *C*-Hexosyl-luteolin *O*-hexoside
141. Isovitexin	142. Luteolin 6-*C*-glucoside	143. 8-*C*-Hexosyl-hesperetin *O*-hexoside	144. Vitexin 2″-*O*-β-l-rhamnoside		
**Hydroxycinnamoyl Derivatives**					
145. *p*-Coumaric acid	146. *trans*-Cinnamaldehyde	147. Hydrocinnamic acid	148. *p*-Coumaraldehyde	149. Caffeic aldehyde	150. 2-Methoxy-benzoic acid
151. Sinapic acid	152. 6-Hydroxymethyl-herniarin	153. Gallic acid *O*-feruloyl-*O*-hexosyl-*O*-hexoside	154. *p*-Coumaryl alcohol	155. Coniferyl alcohol	156. Sinapyl alcohol
157. Caffeic acid *O*-glucoside	158. 3-(4-Hydroxy-phenyl) propionic acid	159. 3,4-Dimethoxy-cinnamic acid	160. Hydroxy-methoxy-cinnamate	161. Cafestol	162. 1-*O*-β-d-Glucopyranosyl sinapate
163. Vanillic acid	164. Coniferin	165. Coniferyl aldehyde	166. Cinnamic acid	167. Ferulic acid	168. Caffeic acid
169. Sinapin-aldehyde	170. 3-Hydroxy-4-methoxycinnamic acid	171. Syringaldehyde	172. Syringin	173. Pinoresinol	174. Syringic acid
175. (+)-Piperitol					
**Quinate and Its Derivatives**					
176. Quinic acid *O*-glucuronic acid	177. Chlorogenic acid (3-*O*-Caffeoyl-quinic acid)	178. 3-*O*-*p*-Coumaroyl shikimic acid *O*-hexoside	179. Eudesmoyl quinic acid	180. 1-*O*-Caffeoyl quinic acid	181. 3-*O*-*p*-Coumaroyl quinic acid *O*-hexoside
182. Neochlorogenic acid (5-*O*-Caffeoyl-quinic acid)	183. 5-*O*-*p*-Coumaroyl shikimic acid *O*-hexoside	184. 3-*O*-*p*-Coumaroyl quinic acid	185. Quinic acid		
**Flavonolignan**					
186. Tricin 4′-*O*-(syringyl alcohol) ether 7-*O*-hexoside					
